# Glass-Transition Dynamics of Mixtures of Linear Poly(vinyl methyl ether) with Single-Chain Polymer Nanoparticles: Evidence of a New Type of Nanocomposite Materials

**DOI:** 10.3390/polym11030533

**Published:** 2019-03-21

**Authors:** Beatriz Robles-Hernández, Marina González-Burgos, José A. Pomposo, Juan Colmenero, Ángel Alegría

**Affiliations:** 1 Departamento de Física de Materiales, University of the Basque Country (UPV/EHU), Apartado 1072, 20080 San Sebastián, Spain; josetxo.pomposo@ehu.eus (J.A.P.); juan.colmenero@ehu.eus (J.C.); 2 Donostia International Physics Center (DIPC), Paseo Manuel Lardizábal 4, 20018 San Sebastián, Spain; 3 Materials Physics Center, CSIC-UPV/EHU, Paseo Manuel Lardizábal 5, 20018 San Sebastián, Spain; marina.gonzalez@ehu.eus; 4 IKERBASQUE—Basque Foundation for Science, María Díaz de Haro 3, E-48013 Bilbao, Spain

**Keywords:** nanocomposites, single-chain polymer nanoparticles, polymer blends, dielectric relaxation, glass transition

## Abstract

Single-chain polymer nanoparticles (SCNPs) obtained through chain collapse by intramolecular cross-linking are attracting increasing interest as components of all-polymer nanocomposites, among other applications. We present a dielectric relaxation study on the dynamics of mixtures of poly(vinyl methyl ether) (PVME) and polystyrene (PS)-based SCNPs with various compositions. Analogous dielectric measurements on a miscible blend of PVME with the linear precursor chains of the SCNPs are taken as reference for this study. Both systems present completely different behaviors: While the blend with the linear precursor presents dynamics very similar to that reported for PVME/PS miscible blends, in the PVME/SCNP mixtures there are an appreciable amount of PVME segments that are barely affected by the presence of SCNPs, which nearly vanishes only for mixtures with high SCNP content. Interestingly, in the frame of a simple two-phase system, our findings point towards the existence of a SCNP-rich phase with a constant PVME fraction, regardless of the overall concentration of the mixture. Moreover, the dynamics of the PVME segments in this SCNP-rich phase display an extreme dynamic heterogeneity, a signature of constraint effects.

## 1. Introduction

Polymer mixing is a convenient method to obtain materials with certain desirable properties, starting from existing materials which do not fit the prescribed requirements. Within this broad range of materials, miscible polymer blends have been widely used for easy tuning of the glass transition temperature ($T_{g}$) of the resulting mixture [1]. There has been intense research into the detailed investigation of the molecular dynamics of miscible polymer blends [2], which generally requires the combination of different experimental techniques; for instance, dielectric spectroscopy (DS) [3], quasi-elastic neutron scattering (QENS) [4], and nuclear magnetic resonance (NMR) [5], among others. Thanks to previous studies, it is now well established that the glass transition of miscible polymer blends extends over a broad temperature range [1] in the interval between the two $T_{g}$ values of the homopolymers. This occurs as a result of the superposition of disparate contributions from each blend component, each related to two distinct segmental relaxation processes, with different characteristics and temperature dependencies [2]. The detailed study of each component requires the use of an experimental technique with selectivity. Broad-band dielectric spectroscopy (BDS) is a suitable technique for mixtures where only one component presents a significant molecular dipole, fluctuating with the segmental time scale [3]. A prototype of such a miscible polymer blend is the system poly(vinyl methyl ether)/polystyrene (PVME/PS) [6,7], where PVME is basically the only dielectrically active component, as the dielectric relaxation strength at the glass transition temperature of PS is much weaker than that of PVME.

Another approach in tuning the material properties, starting from already existing materials, is that of polymeric composites [8]. This is a very promising approach, particularly in the case of polymer nanocomposites where, by means of proper surface treatment of the inorganic fillers, it is possible to obtain good nanoparticle dispersion into the polymer matrix and, consequently, a fine-tuning of the resulting material properties [9]. However, the glass transition of nanocomposite results are, in general, less affected, the major effect being a broadening of the glass transition range towards higher temperatures [10,11]. On the other hand, there has been significant effort towards the development of what has been referred to as single-chain polymer nanoparticles (SCNPs) [12]. SCNPs are obtained through the folding/collapse of individual polymer chains at high dilution (below the overlap concentration) by intra-chain cross-linking reactions [12,13,14,15,16]. For SCNP construction, intramolecular cross-linking techniques based on (i) non-covalent, (ii) covalent, and (iii) dynamic covalent bonds have been developed [17,18,19,20]. The above techniques gave rise to folded/collapsed soft nano-objects with very promising applications in different fields, including catalysis [12,21,22], sensing [12,23,24] and nanomedicine [12,25,26,27,28,29], among other applications.

A recently explored application of SCNPs is the possibility of obtaining all-polymer nanocomposites with unique characteristics, by mixing conventional linear polymer chains with SCNPs. All-polymer nanocomposites based on SCNPs were first reported by Mackay et al. [30,31,32]. Single-chain PS nanoparticles of different sizes were dispersed in linear PS matrices of varying molecular weight and the dispersion degree of the nanoparticles in the resulting all-polymer nanocomposites was determined by small-angle neutron scattering (SANS). PS-SCNPs were also evaluated as useful additives in arresting the phase separation of homogeneous PS/PVME blends showing phase splitting at a high temperature [33]. More recently, the microscopic dynamics of all-polymer nanocomposites composed of single-chain poly(methyl methacrylate) nanoparticles and linear poly(ethylene oxide) have been investigated by neutron spin echo (NSE) experiments, showing a spectacular disentanglement of PEO chain-motions for the all-polymer nanocomposite containing 25% SCNPs [34,35,36,37]. However, when considering the glass transition phenomenon, an open question is how such mixtures of linear polymers with SCNPs compare with conventional polymer-polymer mixtures; namely, with miscible polymer blends [1].

In this work, we have addressed this question by investigating the dynamic behavior around the glass transition of PVME mixtures with PS-based SCNPs by means of BDS, and compared the results with those from a miscible blend of PVME with the linear precursor of the SCNPs. As a main result, we found that the behavior of the PVME/SCNP mixtures was dramatically different from that of the miscible blend. For all compositions, there was a clear signature of the presence of dispersed SCNPs, which incorporated PVME chain segments on a matrix formed by nearly pure PVME. The PVME segments incorporated within the SCNPs presented extremely heterogeneous dynamics, most probably involving chain strands consisting of several repeating PVME units. Irrespective of the overall concentration, it was found that the PVME segments incorporated into the SCNPs amount to about 1/3 of the weight of the SCNP content.

## 2. Materials and Methods

The SCNPs were synthesized by intra-molecular cross-linking of individual linear polymeric chains (precursors) upon a microwave-assisted azide decomposition in DMF solution, according to the procedure reported earlier [38] (see Supplementary Materials). As a linear precursor of the SCNPs, a random copolymer of styrene and 4-(azidomethyl) styrene (AMS), namely P(S${}_{0.7}$-*ran*-AMS${}_{0.3}$), with molecular weight $M_{w}$ = 275 kDa and a polydispersity MwMwMnMn = 1.3 was used. Poly(vinyl methyl ether) (PVME) was obtained from Aldrich Chemicals. PVME/SCNP mixtures with various compositions (PVME weight fractions $\phi_{\mathrm{PVME}}$ of 75%, 50%, and 25%) were prepared by dissolving the two components in tetrahydrofuran (THF) and casting from the solution directly onto electrodes for dielectric relaxation measurements. The blend films were further dried in a vacuum oven at 333 K for 48 h. This drying process assured that the solvent was completely removed and avoided any bubble formation in the sample. The samples will be denoted as “XPVME” throughout the article, where X is the weight percentage of PVME in the sample. A sample containing 50% of PVME, but with linear precursor chains instead of SCNPs, was prepared and investigated in parallel. This blend will be called “50PVME/50Prec”.

### 2.1. Broad-Band Dielectric Spectroscopy

In order to characterize the dielectric relaxation of the mixtures, BDS experiments were carried out by using an Alpha-A Novocontrol dielectric analyzer covering the frequency range $10^{-2}$–$10^{6}$ Hz. Samples were placed between two circular gold-plated brass electrodes with a diameter of 20 mm, forming a parallel plate capacitor. The sample thickness was maintained using a 0.1 mm thick Teflon spacer with negligible area. The temperature was controlled by a nitrogen-jet stream with a Novocontrol Quatro temperature controller. Frequency sweeps were performed at constant temperature, with a stability better than 0.05 K. The temperature range investigated was 120–350 K.

### 2.2. Temperature-Modulated Differential Scanning Calorimetry (TMDSC)

TMDSC measurements were performed on ∼10 mg of the sample, using a Q2000 TA Instruments set-up equipped with a liquid nitrogen cooling system. A helium flow rate of 25 mL/min was used throughout. Measurements were performed by placing the sample in sealed aluminum pans. Data were acquired on cooling at an average rate of 3 K/min, where the temperature oscillated around the average value with 0.5 K amplitude and a 60 s period.

## 3. Results

### 3.1. PVME/SCNP Mixture versus PVME/Prec Blend

**Figure 1:**
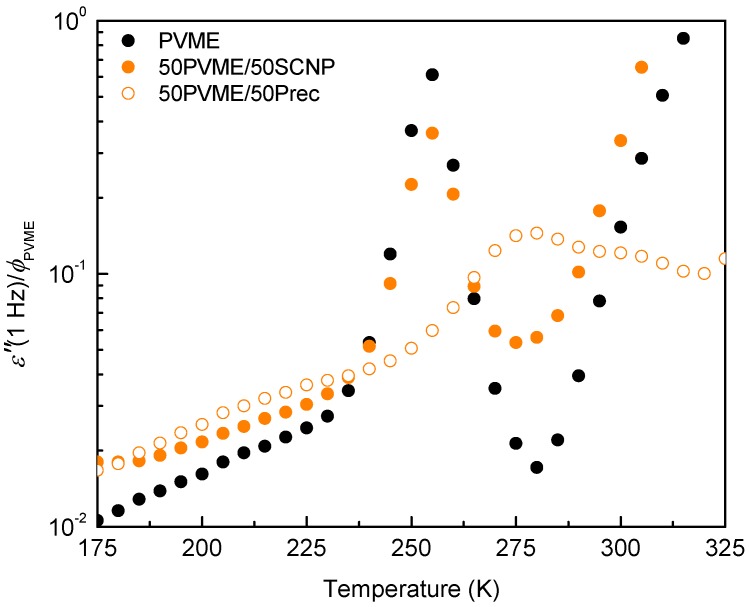
Isochronal *ε*″ $\left(T\right)$, obtained at 1 Hz on pure poly(vinyl methyl ether) (PVME) (black symbols), the 50% mixtures with the single-chain polymer nanoparticles (SCNPs) (full symbols), and with the precursor (empty symbols). For each sample, the *ε*″ values are divided by the PVME weight fraction $\phi_{\mathrm{PVME}}$.

Figure 1 shows the temperature dependence of the dielectric loss *ε*″, measured at 1 Hz on the PVME homopolymer as well as on the 50% mixtures with the precursor and with the SCNPs, respectively. Note that the *ε*″ values were normalized by the PVME weight fraction $\phi_{\mathrm{PVME}}$ to get a relevant comparison of the intensity of the relaxation peaks among the different systems. For each sample, we observe a main loss peak corresponding to the α-relaxation process. In the case of the blend with the linear precursor, the α-relaxation peak is strongly modified with respect to that of neat PVME: The peak shifts to higher temperatures and displays a strong broadening. This behavior is very similar to that observed in the extensively-studied PVME/PS miscible blends [6,7]. The shift of the peak can be related to the slowing down of the PVME segment motions by the presence of stiffer precursor chains. Conversely, in the mixture with the SCNPs there is a decrease in the peak intensity without clear indications of any broadening or shifting of the main relaxation peak.

**Figure 2:**
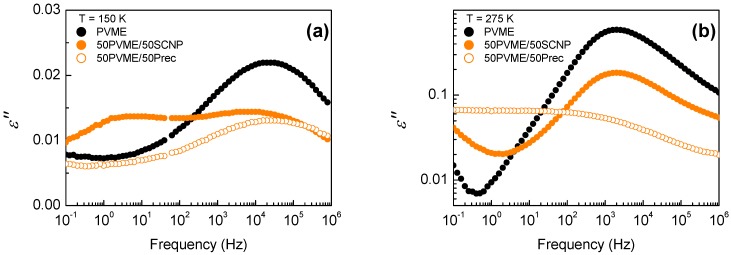
Dielectric loss curves for PVME (black symbols), the 50% mixtures with the SCNPs (full symbols), and the precursor (empty symbols), at a temperature (**a**) below and (**b**) above $T_{g}$.

Figure 2 shows a detailed comparison of the isothermal dielectric losses of PVME homopolymer and the 50 wt% mixtures with the precursor and the SCNPs below and above $T_{g}$. Below $T_{g}$ (Figure 2a), only the secondary processes are observed. Note that the mixture with the SCNPs displays a bimodality, due to the noticeable contribution of the β-relaxation of the SCNPs [38]. From the results shown in Figure 2a, it is apparent that, in both systems, neither the position nor the shape of the β-relaxation loss peak of PVME are modified by blending, beyond the experimental uncertainties. Above $T_{g}$, the differences between the dielectric response of the two mixtures are evident: While the α-relaxation behavior of the linear precursor blend is comparable to that of the PVME blends with neat PS [6,7], in the mixture with the SCNPs there is a decrease of the main peak intensity without significant shifting or broadening.

### 3.2. PVME/SCNP Mixtures

**Figure 3:**
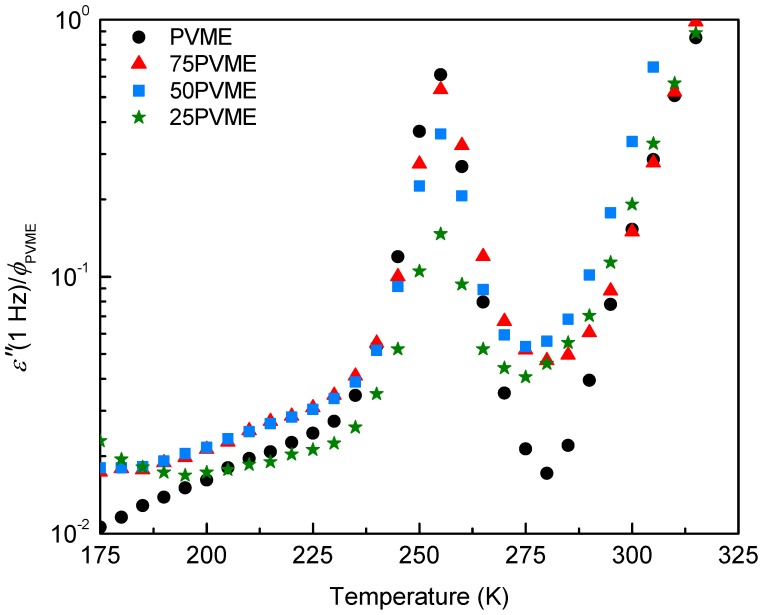
Loss component *ε*″ $\left(T\right)$ of the complex dielectric permittivity obtained at 1 Hz as a function of temperature for pure PVME and PVME/SCNP mixtures with various compositions ($\phi_{\mathrm{PVME}}=75$, 50, and 25 wt%). For each sample, the *ε*″ values are divided by the PVME weight fraction $\phi_{\mathrm{PVME}}$.

Figure 3 shows the dependence on temperature of the dielectric losses (normalized by the PVME weight fraction) at 1 Hz for pure PVME, as well as for the mixtures with the SCNPs at various compositions (75, 50, and 25 wt %). As can be seen, the position and width of the main peak related to the α-relaxation does not change much with the amount of SCNPs in the mixtures. However, the reduction in loss peak intensity is larger than expected, due to the decrease of the PVME content in the samples, and this difference increases as the fraction of PVME decreases.

**Figure 4:**
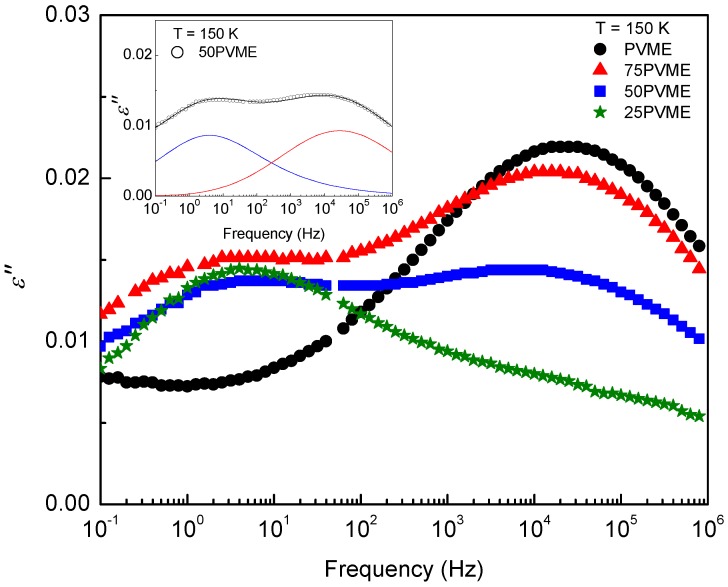
Dielectric loss curves at T=150 K for PVME (black) and PVME/SCNP mixtures, with $\phi_{\mathrm{PVME}}=75\%$ (red), 50% (blue), and 25% (green). Inset: Example of fitting for the 50PVME mixture; the red line corresponds to the β-process from PVME and the blue line to the β-relaxation from SCNPs.

Figure 4 displays the dielectric spectra of the PVME homopolymer and the PVME/SCNP mixtures at 150 K, where only the secondary processes are observed. Although it is not evident forthe 25PVME mixture at the selected temperature in Figure 4, all the mixtures show a bimodality due to the β-relaxation contribution from the SCNPs. There is an interchange of the intensity of the two relaxation components as the PVME fraction decreases. Interestingly, the secondary relaxation of the mixtures can be described by a simple superposition of the β-relaxation of the components:

(1) $$\Phi_{\beta }^{*}=\left(1-\phi_{\mathrm{PVME}}\right)\Phi_{\beta ,\mathrm{SCNPs}}^{*}+\phi_{\mathrm{PVME}}\Phi_{\beta ,\mathrm{PVME}}^{*}.$$

For this analysis, the β-relaxation from PVME was described as a superposition of Debye-like thermally activated processes distributed according to a Gaussian distribution of activation energy barriers [39,40,41], which in the frequency domain reads

(2) $$\Phi_{\beta ,\mathrm{PVME}}^{*}\left(\omega \right)=\int_{0}^{\infty }g\left(E\right)\frac{1}{1+i\omega \tau_{0}\exp\left(\frac{E}{kT}\right)}dE,$$

where the distribution function $g\left(E\right)$ is taken as

(3) $$g\left(E\right)=\frac{1}{\sqrt{2\pi }\sigma_{E}}\exp\left[-\frac{1}{2}{\left(\frac{E-E_{0}}{\sigma_{E}}\right)}^{2}\right],$$

with the parameters taken from [41]. The β-relaxation from SCNPs was characterized by a Cole-Cole function [42],

(4) $$\Phi_{\beta ,\mathrm{SCNPs}}^{*}\left(\omega \right)=\frac{1}{{1+\left(i\omega \tau_{\mathrm{CC}}\right)}^{\alpha }},$$

with the relaxation time taken from [38], and α≃0.6. The inset on Figure 4 shows as an example of the good accuracy provided by Equation (1), together with each of the individual contributions following Equations (2) and (4).

**Figure 5:**
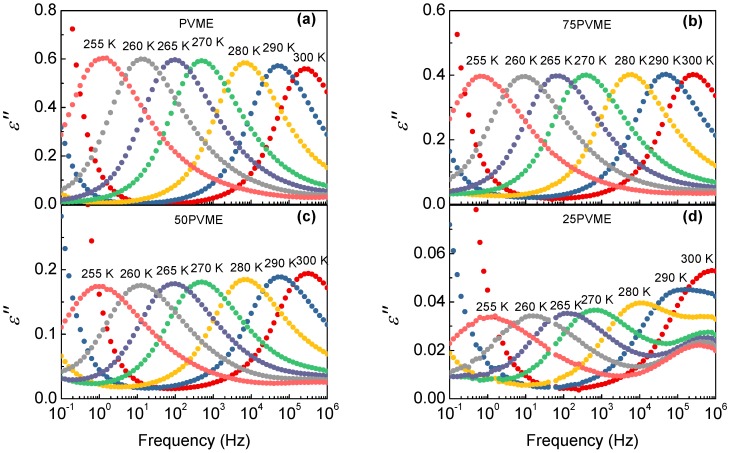
Dielectric loss curves at several temperatures above $T_{g}$ for (**a**) pure PVME and PVME/SCNP mixtures with various compositions: (**b**) $\phi_{\mathrm{PVME}}=75\%$, (**c**) 50%, and (**d**) 25%.

A comparison of the dielectric relaxation behavior of PVME with those of the mixtures above $T_{g}$ is shown in Figure 5. A main loss peak, corresponding to the PVME segmental dynamics, is detected, along with a much weaker higher frequency signal related to the secondary relaxation processes. Concerning the comparison of the α-relaxations in the different samples, there are clear changes in the peak intensity but the differences in the widths and the peak frequencies are minor. Note that, as the PVME fraction decreases, the contribution from the secondary relaxation from the SCNPs is more noticeable and superimposes more with the α-relaxation, mainly at higher temperatures (see Figure 5d).

**Figure 6:**
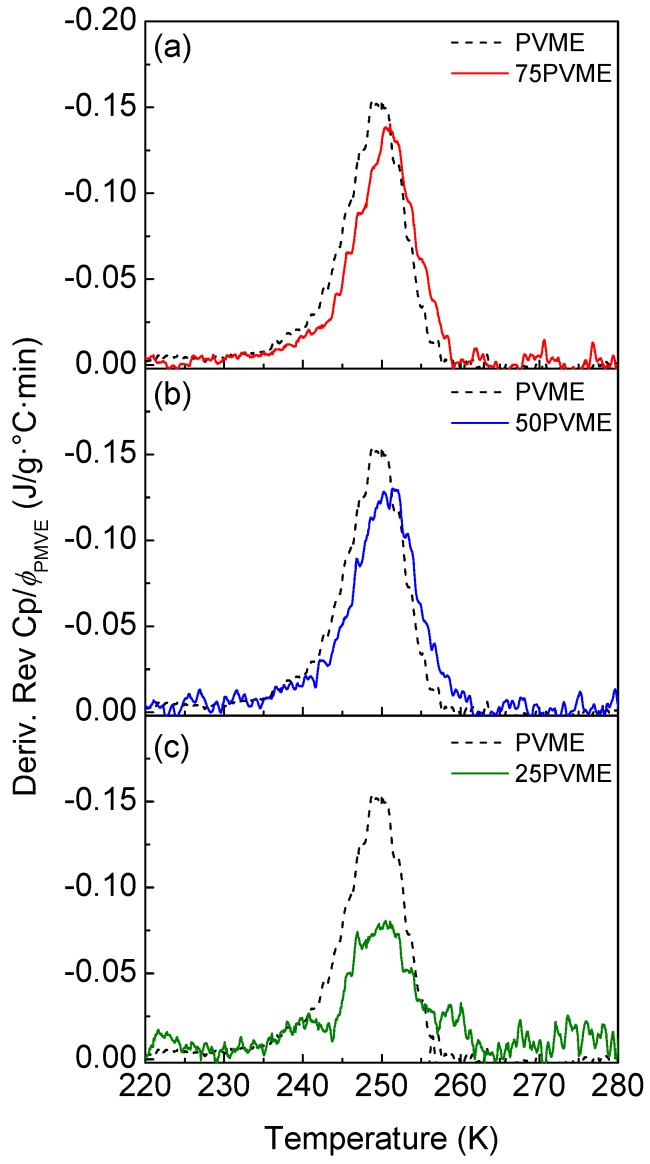
Derivative of the reversing heat capacity (normalized by the PVME weight fraction $\phi_{\mathrm{PVME}}$) for PVME and the mixtures: (**a**) 75PVME, (**b**) 50PVME, and (**c**) 25PVME.

Figure 6 shows the results obtained by TMDSC on the glass transition of the investigated PVME/SCNP mixtures, as compared with the corresponding one for neat PVME. The comparison shows that the reversing heat capacity derivative in the mixtures presents a clear peak around the glass transition temperature of neat PVME. However, the transition range is slightly broader in the mixtures, which is indicative of the presence of slower components. Both findings are in agreement with the BDS results presented above, if one takes into account that the SCNP contribution to the TMDSC curves is not negligible, but is for the corresponding BDS loss curves.

## 4. Discussion

**Figure 7:**
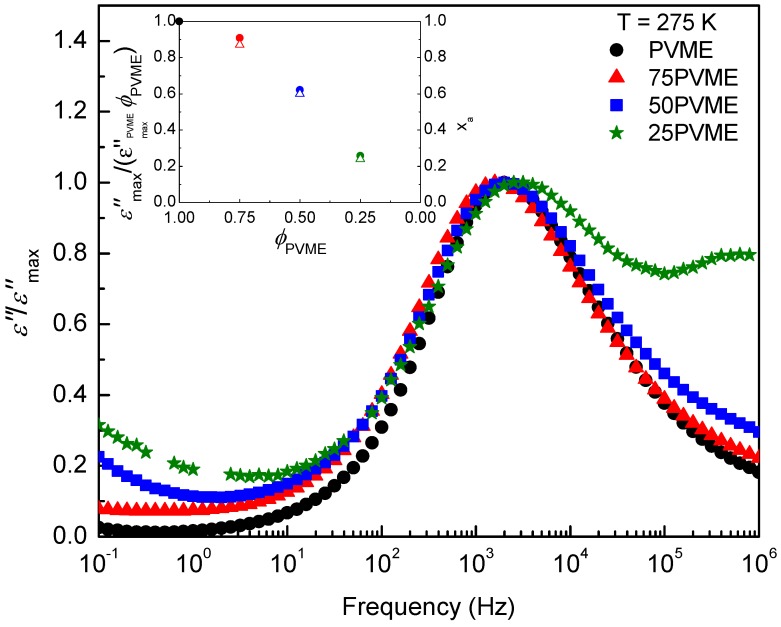
Dielectric loss curves (normalized by the PVME weight fraction $\phi_{\mathrm{PVME}}$), recorded at T=275 K, on PVME and PVME/SCNP mixtures with $\phi_{\mathrm{PVME}}=75\%$ (red), 50% (blue), and 25% (green). Inset: Maxima of the loss peak, $\varepsilon_{\max}^{\prime \prime }$ (divided by the PVME weight fraction $\phi_{\mathrm{PVME}}$), as a function of the PVME weight fraction.

To gain insight into the existing differences among the dielectric α-relaxation behavior of the mixtures and neat PVME, the normalized loss peaks at 275 K for PVME and for the mixtures are represented in Figure 7. The inset in Figure 7 shows the intensity of the corresponding loss peaks, normalized by the PVME concentration $\phi_{\mathrm{PVME}}$, as a function of PVME concentration. It is noticeable that the resulting values are lower than the ones for the mixtures, an indication of a lack of PVME dipoles contributing to the main loss peak frequency. This effect increases as the PVME fraction in the mixture decreases. When comparing the normalized curves, it is found that, as the PVME fraction decreases, a high frequency broadening, which is directly attributable to the presence of a secondary relaxation from the SCNPs, is observable. Besides, there is a lower frequency signal which increases with the concentration of SCNPs, which would be related with the slower motions of the PVME segments in the mixtures.

**Figure 8:**
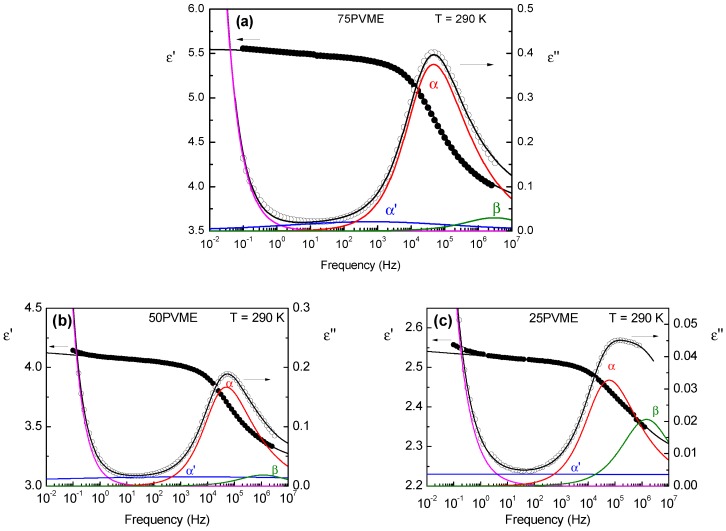
Dielectric spectra of (**a**) 75PVME, (**b**) 50PVME, and (**c**) 25PVME at T=290 K. Solid black lines are the fitting curves obtained by the addition of α-relaxation (red), an additional slow process (blue), β-relaxation (green), and DC-conductivity contribution (magenta).

**Figure 9:**
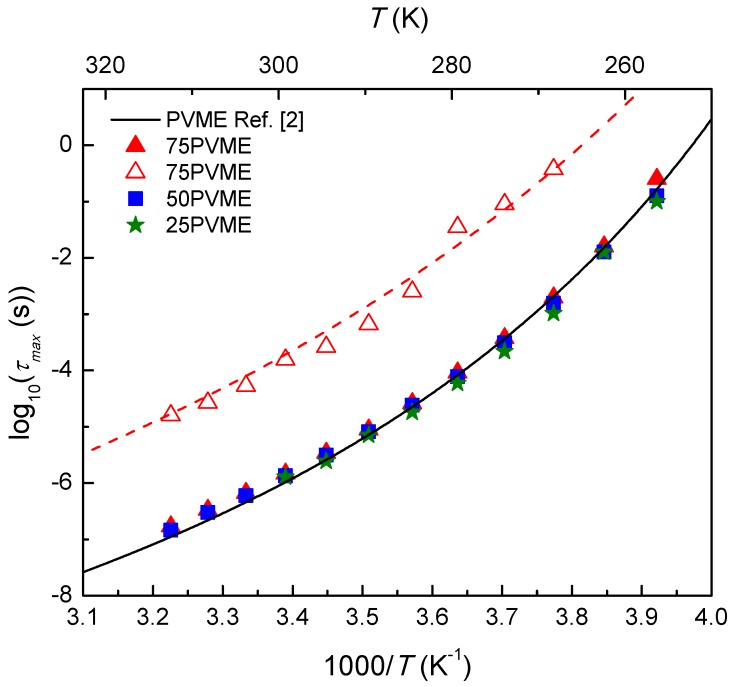
Arrhenius plot of the characteristic relaxation times for the PVME/SCNP mixtures as a function of temperature. The solid line corresponds to a description of the data of PVME homopolymer by means of the Voguel-Fulcher-Tammann (VFT) equation [3]. Full Symbols correspond to the relaxation of the main component of the mixtures ($\phi_{\mathrm{PVME}}=75\%$ (red), 50% (blue), and 25% (green)) and empty symbols to the relaxation of the slow component in the 75PVME mixture. The dashed line represents the temperature dependence obtained from the rheological experiments on neat PVME.

For the analysis of the PVME/SCNP data, the shape parameters of the main contribution of the segmental relaxation can be fixed to those determined from the PVME homopolymer fitting. An additional function was needed to account for the lower frequency contributions, which was assumed to be of the Cole-Cole type. Therefore, the loss curve of the PVME/SCNP mixtures was fitted using the following equation:

(5) $$\varepsilon \prime \prime \left(\omega \right)=\phi_{\mathrm{PVME}}\Delta \varepsilon_{\alpha }\left[x_{a}Im\left\{-\Phi_{\alpha }^{*}\left(\omega \right)\right\}+\left(1-x_{a}\right)Im\left\{-\Phi_{\mathrm{CC}}^{*}\left(\omega \right)\right\}\right]+Im\left\{-\Phi_{\beta }^{*}\left(\omega \right)\right\}+\frac{\sigma_{\mathrm{DC}}}{\omega },$$

where $\Phi_{\beta }^{*}\left(\omega \right)$ is given by Equation (1), $\Phi_{\alpha }^{*}\left(\omega \right)$ corresponds to neat PVME α-relaxation function, the dielectric relaxation strength $\Delta \varepsilon_{\alpha }$ is fixed to that determined for neat PVME, $\sigma_{\mathrm{DC}}$ corresponds to the direct current (DC) conductivity, and $x_{a}$ would represent the fraction of PVME segments in the mixture barely affected by the presence of SCNPs. The solid lines in Figure 8 correspond to the different contributions. As expected from the direct data comparison, the peak frequencies of the PVME-like components overlap well with that of neat PVME (see Figure 9). Moreover, the corresponding fractions of PVME segments in the mixtures that are barely affected by the presence of SCNPs are close to the ratio of the loss peak intensities presented above (see inset in Figure 7). Thus, it can be confirmed, that when increasing the SCNP amount, the fraction of PVME segments strongly influenced by the SCNPs rapidly increases. On the contrary, for low SCNP amounts, there is a large fraction of PVME segments with a dynamic behavior indistinguishable from that of pure PVME. A rough calculation, based on a simple two-phase picture, would indicate that, for 75PVME, there exists a SCNP-rich phase with about 28 wt % PVME. For 50PVME, the SCNP-rich phase would contain about 28 wt % PVME and, for 75PVME, the SCNP-rich phase would contain about 24 wt % PVME. This finding can be interpreted as indicative of the fact that the SCNPs can incorporate a maximum amount of PVME segments. From the previous values, we estimate the PVME amount that is actually incorporated into the SCNPs in any mixture to be about 33 wt % of the SCNPs weight.

Now, focusing our attention on the dynamics of the PVME segments markedly affected by the presence of the SCNPs, we found a highly heterogeneous behavior already for the 75PVME mixture. For this concentration, the Cole-Cole component (see Figure 8a) is very broad, but still presented a reasonable well-defined peak frequency, which was about two decades lower than that of the neat PVME α-relaxation peak. The corresponding relaxation time values are included in Figure 9. Note that the temperature dependence of the slower relaxation component hardly converged at high temperatures with that of the main relaxation peak, which suggests that the molecular entities responsible for this slower relaxation component were considerably larger than the size of the PVME segments responsible for the conventional α-relaxation of PVME. A possible reason for the contribution to the dielectric relaxation of relatively large PVME chain-strands would be the fact that, in the mixtures, some PVME chain portions could be highly constrained inside of the essentially rigid SCNPs. In such a situation, one could expect that the motion of relatively large chain-strands is required for the complete reorientation of the repeating unit dipole moment. Guided by this idea, we have compared the temperature dependence of the dielectric slow component with that of the terminal mechanical relaxation of pure PVME (see Figure 9, dashed line), which included the contributions of long PVME chain-strands. In this way, we found that the temperature dependence of the slower dielectric component in the mixtures was actually well-described with the mechanical shift factor in the terminal region of neat PVME, which supports the aforementioned interpretation.

The mixtures with a higher amount of SCNPs presented an even more heterogeneous slow component. Already for the 50PVME mixture, the CC component accounting for the affected PVME segments became extremely broad, without any well-defined characteristic time and with significant contributions, also, at frequencies well above that corresponding to the barely-affected PVME segments. This finding can be considered as a signature of the onset of evident effects associated to constraints, resembling those observed in nanostructured systems, such as semicrystalline polymers or segregated diblock copolymers [43]. The presence of faster segmental motions in these systems has been interpreted as originated by packing frustration, produced by the constraints on the mobile segments from the presence of rigid nanostructures which, in the present case, would be the relatively stiffer SCNPs. At a high enough concentration, the SCNPs would overlap each other, imposing more dramatic constraints on the trapped PVME chain strands than those already present in the 75PVME mixture. This idea is in good agreement with that recently inferred from molecular dynamic simulations [44].

Comparing the previous findings with the TMDSC results should facilitate establishing the consequences of the dynamical peculiarities of the PVME/SCNP mixtures on the glass transition phenomenon. The broader glass transition range, found in the mixtures, can be directly connected with the presence of the slower component of the α-relaxation. For instance, for 75PVME, the reversing heat capacity derivative peak extends up to about 260 K (see Figure 6a). Interestingly, at 260 K, the relaxation time of the slower component identified in the dielectric relaxation takes a value close to that of the neat PVME at the temperature where the TMDSC peak vanishes, consistent with the proposed interpretation. On the other hand, by comparing the area of the two TMDSC peaks in Figure 6a, it seems that the thermal glass transition in the 75PVME mixture is nearly completed at 260 K; although a remaining (but very weak) higher temperature signal would exist. The 50PVME mixture shows a more pronounced peak shift and a more extended high temperature flank (see Figure 6b), indicative of more pronounced effects. Additionally, a remaining (but weak) higher temperature signal would exist above 270 K. Finally, the 25PVME mixture shows a clearly reduced peak intensity in the glass transition range of neat PVME, indicative of a much smaller fraction of unaffected PVME segments, which is what was found by the BDS data analysis. In this mixture, the remaining signal associated with the thermal glass transition would be very much extended towards high temperatures, being, however, indistinguishable from the background noise (see Figure 6c). In fact, the contributions from the PS segments to the TMDSC signal should eventually exist at higher temperatures.

When the results obtained in the mixtures of PVME with SCNPs are compared with those of PVME mixtures with linear PS chains [6,7], the observed differences were dramatic. Whereas the latter follow the common behavior generally observed for other miscible polymer blends, the mixtures of PVME with SCNPs present a glass-transition phenomenology that is closer to that widely reported for polymer nanocomposites [10,11]. In this view, a matrix formed by linear chains would exist; being the fillers formed by the SCNPs penetrated with PVME chain portions. This structure would resemble that of polymer-functionalized inorganic nanofillers, where functionalization is used both for improving the interaction between the polymer matrix and the nanoparticles, and to prevent nanoparticle agglomeration. Therefore, the mixtures of linear polymers and miscible SCNPs give rise to a new type of composite materials, with a glass transition behavior that is closer to that of conventional nanocomposites than to that usually observed in miscible polymer blends.

## 5. Conclusions

We have investigated the dynamics of the PVME segments in mixtures of PVME and polystyrene-based SCNPs with several concentrations by means of BDS, taking, as reference, a PVME blend with the linear precursor chains of the SCNPs. Two main relaxation processes were observed: Secondary β-relaxation and segmental α-relaxation. The local motions responsible for the β-relaxation are not affected by the mixing in either system. On the other hand, we have found a strikingly different behavior for the segmental dynamics of the mixtures with the SCNPs, as compared with a mixture using the linear precursor. In the latter blend, the α-relaxation is strongly affected by blending, just like in the miscible blends of PVME with PS. On the contrary, in the mixtures of PVME with SCNPs, there is a limited fraction of PVME segments dramatically affected and the rest present a dynamic behavior nearly identical to that of pure PVME. Our findings show that the dynamics of the PVME segments affected by the presence of stiffer SCNPs are highly heterogeneous, as found already in PVME-rich mixtures. Moreover, in the mixtures with a higher concentration of SCNPs, the heterogeneity further increases and there are indications that, in the mixture, some PVME segments move faster than in neat PVME, which is associated to constraint effects. Furthermore, our results show that there is a limit to the amount of PVME segments that can be incorporated into close contact with the SCNPs, which amounts to about 1/3 in weight. Overall, our results confirm that mixtures of linear polymers and SCNPs constitute a new type of promising nanocomposite materials.

## Supplementary Materials

The following are available at https://www.mdpi.com/2073-4360/11/3/533/s1.
