# Supplementary material for: Glass-Transition Dynamics of Mixtures of Linear Poly(vinyl methyl ether) with Single-Chain Polymer Nanoparticles: Evidence of a New Type of Nanocomposite Materials
Source: Polymers (Basel). 2019 Mar 21;11(3):533. doi: 10.3390/polym11030533 (PMC6473516; doi:10.3390/polym11030533)
Supplement: Supplementary file 1 [file polymers-11-00533-s001.pdf]

Supplementary Materials: Glass-Transition Dynamics of Mixtures of Linear Poly(vinyl methyl ether) with Single-Chain Polymer Nanoparticles: Evidence of a New Type of Nanocomposite Materials

Beatriz Robles-Hernández [1,2,*], Marina González-Burgos [3], José A. Pomposo [1,3,4], Juan Colmenero [1,2,3] and Ángel Alegría [1,3,*]

1. Materials

Styrene (S) ($\geq$ 99%), 4-(chloromethyl) styrene (CMS) ($\geq$ 90%), sodium azide (NaN_3) ($\geq$ 99%), dansylhydrazine ($\geq$ 98%), 4,4′-Azobis(4-cyanovaleric acid) (ACVA) ($\geq$ 98%), N-N-Dimethylformamide (DMF) ($\geq$ 99.9%), benzaldehyde (BA) (purified by redistillation, $\geq$ 99.5%), chloroform ($CHCl_3$) ($\geq$ 99.8%) and deuterated chloroform ($CDCl_3$) (99.96 atom % D, containing 0.03% (v/v) tetramethylsilane, TMS) were obtained from Aldrich and used, unless specified, as received. Methanol (MeOH) (synthesis grade) and tetrahydrofuran (THF) (HPLC grade) were purchased from Scharlab. Benzyl azide (94%) was purchased from Alpha Aesar. Deionized water obtained from a Thermo Scientific apparatus (Barnstead TII Pure Water System) was used in this work. S and CMS were purified by passing through basic alumina. Scheme 1 displays the structures of some compounds used in the current investigation.

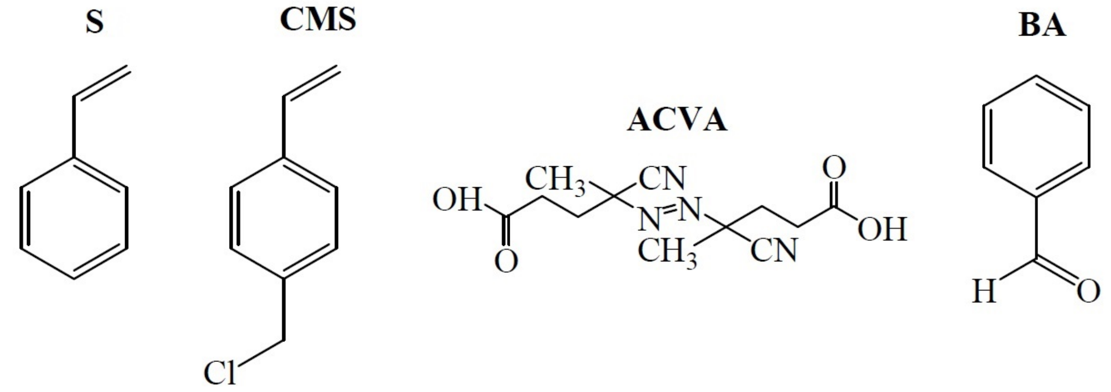

Scheme 1. Structures of some compounds used in the current investigation.

2. Instrumentation

2.1. Microwave Assisted Synthesis

Microwave-assisted synthesis was carried out in a CEM Discover LabMateTM apparatus. It was equipped with an 80 ml Sealed Vessel Accessory, which has a working volume ranging from 15 ml to 50 ml.

3. Synthesis

3.1. Synthesis of poly(styrene-ran-chloromethyl styrene) precursor

In a typical procedure of synthesis of precursor, the copolymerization of S (2 ml, 17.4 mmol) and CMS (0.615 ml, 3.7 mmol) was carried out using ACVA (1.7 mg, 6.1 $\times$ 10$^-$3 mmol) as initiator. The reaction mixture was degassed by passing argon for 15 min and then stirred for 3 h at 353 K. After that,

the copolymer was redissolved in a minimal amount of THF and added to a large excess of methanol. The resulting precursor (C30) was isolated by filtration and further dried at room temperature under dynamic vacuum (Yield: 30%, M_w (size exclusion chromatography, SEC) = 267 kDa, PDI (SEC) = 1.3). Copolymer was obtained as light yellow powders with 30 mol % of CMS content, as determined by ^{1}H-NMR spectroscopy following reported procedures[1]. Scheme 2 summarizes the synthetic route for obtaining the precursor.

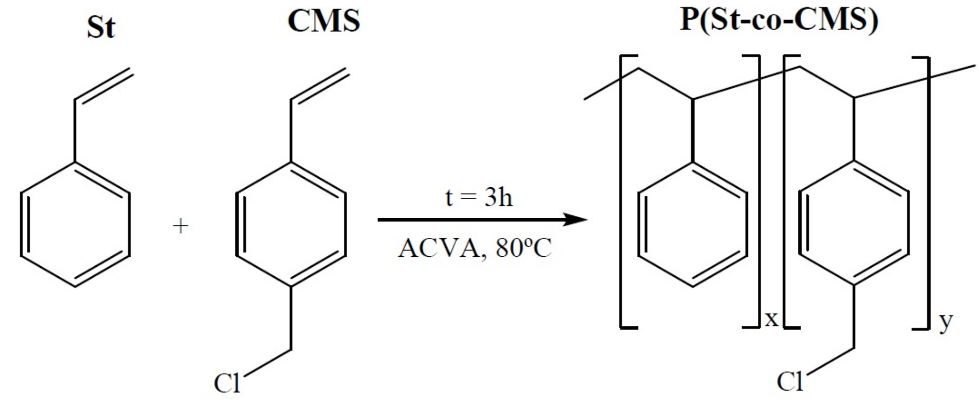

Scheme 2. Synthesis of poly(styrene$_{0.7}$-*ran*-chloromethyl styrene$_{0.3}$) copolymers (y = 0.3 mol).

3.2. *Synthesis of poly(styrene-ran-azidomethyl styrene) precursor*

In a typical reaction, poly(styrene-co-chloromethyl styrene) (C30, 350 mg, 0.64 mmol CMS) was dissolved in DMF (14 ml) at room temperature. Then, NaN_3 (2 eq., 83.2 mg, 1.3 mmol) was added and the mixture was maintained under stirring for 24 h. After reaction completion, the system was concentrated and precipitated in a mixture of MeOH/H_2O (1:1). Finally, the resulting precursor (P30) was dried in a vacuum oven at room temperature under dynamic vacuum (Yield: 90%, M_w (SEC) = 275 kDa, PDI (SEC) = 1.3, R_H (dinamic light scattering, DLS) = 14 nm, T_g (differential scanning calorimetry, DSC) = 366 K). Precursors was obtained as powders with 30% mol of AMS content. The complete transformation of chloromethyl (4.5 ppm) to azidomethyl (4.2 ppm) moieties was confirmed by ^{1}H-NMR spectroscopy (see S.I. in ref.[2]).

3.3. *Synthesis of intra-chain cross-linked nanoparticles*

In a typical reaction, the precursor (P30, 50 mg, 0.432 mmol) was dissolved in DMF (50 mL) at room temperature. Then, the mixture was heated to 473 K under microwave irradiation (300 W, 150 psi) and maintained there for 30 min. Then, the system was cooled down to room temperature and concentrated in a vacuum line using Schlenk flasks. The resulting nanoparticles (NP30) were isolated by precipitation in a mixture of MeOH/H_2O (1:1) and dried in a vacuum oven at 313 K under dynamic vacuum (Yield: 57%, M_w (SEC) = 274 kDa, PDI (SEC)= 1.3, R_H (DLS) = 10 nm, T_g (DSC) 405 K).

References

1. Huang, K.W.; Kuo, S.W. High-Heteronucleobase-Content Polystyrene Copolymers Prepared Using Click Chemistry Form Supramolecular Structures With Melamine Through Complementary Multiple Hydrogen-Bonding Interactions. *Macromol. Chem. Phys.* **2012**, *213*, 1509–1519. doi:10.1002/macp.201200114.
2. González-Burgos, M.; Alegría, A.; Arbe, A.; Colmenero, J.; Pomposo, J.A. An unexpected route to aldehyde-decorated single-chain nanoparticles from azides. *Polym. Chem.* **2016**, *7*, 6570–6574. doi:10.1039/c6py01602b.
